# A Targeted Blockade of Terminal C5a Is Critical to Management of Sepsis and Acute Respiratory Distress Syndrome: The Mechanism of Action of Vilobelimab

**DOI:** 10.3390/ijms26199628

**Published:** 2025-10-02

**Authors:** Matthew W. McCarthy, Camilla Chong, Niels C. Riedemann, Renfeng Guo

**Affiliations:** 1 Department of Medicine, Weill Cornell Medicine, New York, NY 10065, USA; mwm9004@med.cornell.edu; 2 InflaRx GmbH, 07745 Jena, Germanyniels.riedemann@inflarx.de (N.C.R.); 3 InflaRx Pharmaceuticals, Ann Arbor, MI 48103, USA

**Keywords:** complement, extrinsic pathway, C5, C5a, C5b, membrane attack complex, sepsis, acute respiratory distress syndrome, mechanism, vilobelimab

## Abstract

Vilobelimab, a first-in-class, human–mouse chimeric immunoglobulin G4 (IgG4) kappa monoclonal antibody, targets human complement component 5a (C5a) in plasma. Unlike upstream complement inhibitors, vilobelimab does not inhibit the generation of the membrane attack complex (C5b-9), necessary to mitigate certain infections. C5a is a strong anaphylatoxin and chemotactic agent that plays an essential role in both innate and adaptive immunity. Elevated levels of C5a have been associated with pathologic processes, including sepsis and inflammatory respiratory disorders such as acute respiratory distress syndrome (ARDS). Blocking C5a with vilobelimab has shown therapeutic promise. A randomized, multicenter placebo-controlled Phase III study of vilobelimab in patients with severe COVID-19 (PANAMO) found that patients treated with vilobelimab had a significantly lower risk of death by day 28 and 60. Based on this study, the United States Food and Drug Administration (FDA) issued an Emergency Use Authorization (EUA) for Gohibic^®^ (vilobelimab) injection for the treatment of COVID-19 in hospitalized adults when initiated within 48 h of receiving invasive mechanical ventilation (IMV) or extracorporeal membrane oxygenation (ECMO). In January 2025, the European Commission (EC) granted marketing authorization for Gohibic^®^ (vilobelimab) for the treatment of adult patients with severe acute respiratory syndrome coronavirus 2 (SARS-CoV-2)-induced ARDS who are receiving systemic corticosteroids as part of standard of care and receiving IMV with or without ECMO. Herein, we review the mechanism of action of vilobelimab in selectively inhibiting C5a-induced inflammation, outlining its bench-to-bedside development from the fundamental biology of the complement system and preclinical evidence through to the clinical data demonstrating its life-saving potential in the management of COVID-19–induced ARDS.

## 1. Introduction

The innate immune system plays a crucial role as the first barrier to infection, orchestrating early pathogen recognition and clearance through mechanisms such as phagocytosis, complement activation and the release of pro-inflammatory cytokines [1]. Importantly, these actions of the innate immune response, together with the presentation of antigen to T lymphocytes, are essential to the establishment of the adaptive immune response [2]. However, while essential for containing and clearing invading pathogens before the adaptive immune response is fully mobilized, excessive innate immune activation can lead to immune dysregulation and severe systemic inflammation and the development of critical conditions such as sepsis and acute respiratory distress syndrome (ARDS), respectively [3].

Sepsis, acute lung injury, and ARDS share common underlying etiologies, characterized by immune dysregulation, hyperinflammation, and endothelial dysfunction. Neutrophil overactivation with subsequent neutrophil extracellular trap formation (NETosis) leads to the release of reactive oxygen species and granular enzymes which fuel additional inflammatory cascades and activate coagulation pathways [4]. Overactivation of neutrophils is also implicated in a range of other diseases, including small vessel vasculitis and severe dermatological conditions [5,6,7].

The global burden of sepsis and ARDS is substantial. For sepsis, one of the most comprehensive clinical analyses to date, conducted for the Global Burden of Disease Study, found there were 48.9 million cases of sepsis in 2017, and 11 million sepsis-related fatalities [8]. The incidence of ARDS varies significantly across countries, ranging from 10.1 to 78.9 cases per 100,000 people per year, with prevalence comprising 7.1% to 19% of ICU admissions, depending on population and study methodology [9,10]. In the United States, ARDS incidence is estimated to be 64–79 cases per 100,000 people per year [11], whereas a large Spanish epidemiological study of mechanically ventilated ARDS found an incidence of 2.96–20.14 per 100,000 patients per year between 1 January 2000 and 31 December 2022 [12]. Mortality rates also vary globally, ranging from 38 to 55% [12,13].

Dysregulation of the complement system plays a critical role in the development of sepsis and ARDS. The complement system is an enzymatic cascade consisting of over 30 plasma proteins and glycoproteins critical for host defense and inflammation, and is a core component of the innate immune system [14,15,16]. Activation of the complement system enhances the ability of antibodies and phagocytic cells to clear microbes and damaged cells from an organism, promotes inflammation to attract additional phagocytes, and directly attacks pathogens’ cell membranes through activation of the cytolytic membrane attack complex (MAC; C5b-9). Importantly, complement activation leads to the generation of complement factor 5a (C5a), a potent pro-inflammatory anaphylatoxin that promotes chemotaxis via binding to the C5a receptor 1 (C5aR1) on neutrophils, phagocytes, and mast cells at the site of inflammation [17]. C5a is generated through conventional complement pathway activation (classical, lectin and alternative) as well as by direct cleavage of C5 by proteolytic enzymes such as, but not limited to, thrombin and plasmin. This enzymatic cleavage of C5 to produce C5a has been described as the “extrinsic pathway,” which is independent of the conventional complement pathways [18]. Excessive production of C5a is thought to play a critical role in the development and progression of sepsis (bacterial, viral, and other causes) and ARDS [19,20,21].

Given the significant role of the complement system in immuno-inflammatory diseases, pharmacological interventions have been developed to target components of the C5a/C5aR1 signaling axis. Clinical studies in COVID-19, summarized herein, suggest that blocking C5a may be more effective than inhibiting upstream complement components (such as C3 or C5) and allows for tighter control of C5a/C5aR1 axis-driven inflammation, leading to better safety and efficacy outcomes. For example, selective targeting of C5a, versus broader C5 inhibition, preserves MAC formation (and thus mitigates the risk of infection), but also allows for more complete inhibition of C5a produced by both the conventional complement and extrinsic pathways [22]. Inhibition of MAC formation with C5 inhibitors can also potentially lead to an increased risk of bacterial infections.

Vilobelimab is a first-in-class monoclonal antibody that selectively targets C5a, blocking its pro-inflammatory activity [23,24]. Vilobelimab has been extensively studied in pre-clinical models of both sepsis and ARDS, and is the only drug to have demonstrated a significant reduction in mortality and a favorable safety profile in patients with COVID-19–induced ARDS requiring invasive mechanical ventilation (IMV) and extracorporeal membrane oxygenation (ECMO) in a properly powered and placebo-controlled study [25]. In addition, vilobelimab has been investigated in neutrophil-driven disorders such as hidradenitis suppurativa [26] and ANCA-associated vasculitis [27], where it showed signals of clinical efficacy. These findings support the broader therapeutic relevance of C5a blockade in diseases marked by excessive neutrophil activation and tissue injury, extending its potential application beyond viral ARDS to a wider spectrum of neutrophil-mediated inflammatory conditions.

In this review, the bench-to-bedside approach in the development of Gohibic^®^ (vilobelimab) is discussed, outlining the mechanism of action of vilobelimab and supportive preclinical models as well as clinical data in COVID-19-induced ARDS, leading to a Food and Drug Administration (FDA) Emergency Use Authorization (EUA) in the United States and European Medicine Agency (EMA) approval in Europe. The differentiation of blocking C5a versus upstream C5 is discussed as an important advancement in treating sepsis and ARDS with a potentially better safety profile.

## 2. Complement Activation

**Figure 1:**
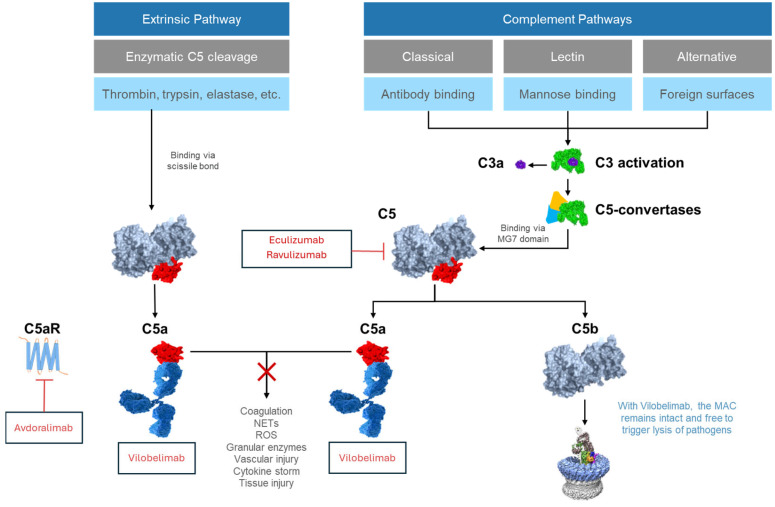
Complement activation pathways and the mechanism of action of vilobelimab. Classical, lectin, and alternative complement activation pathways converge on the generation of active complement split products and the MAC. In this pathway, C5 convertases bind via the MG7 domain of C5, cleaving C5 into its components, C5a and C5b. The extrinsic pathway represents an additional route of complement activation. In this pathway, proteolytic enzymes such as thrombin or plasmin bind to and cleave C5 at or near its scissile bond, to generate C5a. Overactivation of C5a amplifies the innate immune response, contributing to cytokine storm, tissue damage, and multi-organ dysfunction. Vilobelimab blocks C5a generated through both the extrinsic pathway as well as conventional complement pathways, interrupting this inflammatory cascade, while also allowing C5b to be generated as part of MAC formation. By interrupting the inflammatory cascade (X), vilobelimab presumably decreases coagulation, NET formation, ROS generation, granular enzymes release, vascular injury, the cytokine storm, and tissue injury. MAC graphic adapted from Serna et al. [29] licensed by CC BY 4.0. MAC, membrane attack complex; NETs, neutrophil extracellular traps; ROS, reactive oxygen species.

Activation of complement via three conventional pathways—the classical, lectin, and alternative pathways—has been well documented (Figure 1) [22,28]. All three complement pathways converge at the level of C3, which can be enzymatically cleaved/activated by C3 convertases resulting in the formation of the anaphylatoxin C3a and the opsonin C3b. Once a stable C3 convertase complex is formed, it combines with C3b to form the enzyme C5 convertase, which cleaves C5 into its components, C5a and C5b. C5b then recruits C6, C7, C8 and multiple C9 molecules to assemble the MAC that can cause microbial cell lysis and neutralization of viral particles. However, further studies have shown that complement activation extends beyond these well-known complement pathways. Despite being characterized for decades, the role of the extrinsic pathway is often overlooked. In this pathway, proteolytic enzymes such as neutrophil elastase, thrombin, kallikrein, and trypsin can directly cleave C5 to produce C5a independent of the conventional complement cascade, which is particularly relevant during conditions of severe systemic inflammation and/or coagulopathy [18,28].

## 3. Downstream Effects of Activation of the C5a/C5aR1 Axis

**Figure 2:**
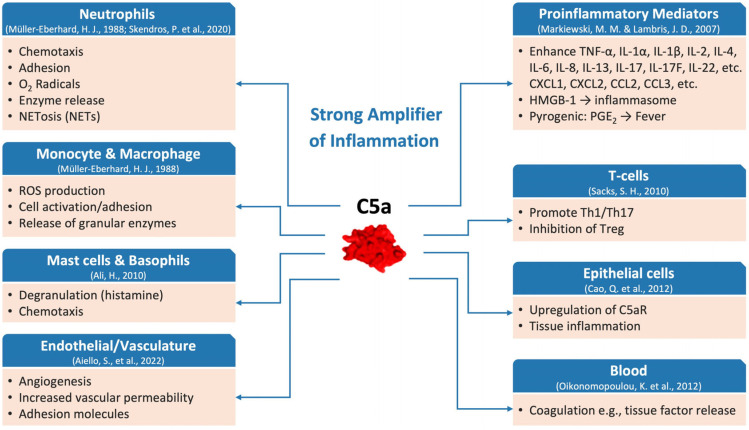
C5a as a central amplifier of inflammation. C5a is a potent pro-inflammatory mediator that activates neutrophils and other immune cells such as monocytes and macrophages. It drives the release of ROS, granular enzymes, NETs, and promotes coagulation, increased vascular permeability, endothelial adhesion, and angiogenesis. CCL, CC motif chemokine ligand; CXCL, CXC motif chemokine ligand; HMGB, high-mobility group box; IL, interleukin; NET, neutrophil extracellular trap; PGE, prostaglandin E; Th, T helper; TNF, tumor necrosis factor [28,34,35,36,37,38,39,40].

C5a exerts its biological effects primarily by binding to C5aR1, a G protein-coupled receptor expressed on a broad range of innate immune cells. Activation of C5aR1 converts complement activation into cellular responses such as chemotaxis, activation, and release of inflammatory mediators. Consequently, the C5a–C5aR1 axis functions as a central mediator of inflammatory responses. (Figure 2) [30,31]. A second C5a receptor (C5aR2, also known as C5L2) has also been implicated in multiple pathways, although it is thought to have a complex regulatory role, possibly influencing C5aR1 receptor trafficking and cytokine expression and secretion [32,33].

C5a is a highly potent inflammatory mediator that significantly contributes to the activation and/or chemotaxis of neutrophils, monocytes, macrophages, mast cells, and smooth muscle cells. It elicits cellular responses such as cell adhesion, chemotactic migration, enzyme release, and the production of arachidonic acid metabolites and reactive oxygen species (ROS) [34]. C5a is also a powerful stimulator of gene expression of multiple inflammatory cytokines, including interleukin (IL)-8, IL-6, IL-17, and Tumor Necrosis Factor (TNF)-α. Excessive C5a levels have been shown to result in apoptosis of thymocytes through a mitochondria-dependent pathway involving caspase-3, -6, and -9 activation and downregulation of Bcl-XL, contributing to an immunosuppressed phenotype [41]. In addition, C5a influences T-cell immune responses by upregulating costimulatory molecules (such as CD40 and CD80) and promoting dendritic cell-mediated activation of Th1 responses [38]. The absence of C5a may result in reduced Th1 responses and potentially altered Th17 and regulatory T cell (Treg) development, shifting immune balance toward Th2 or regulatory phenotypes depending on the inflammatory context [38]. It has also been shown that C5a can directly link innate immunity to coagulation pathways by inducing tissue factor (TF) release from neutrophils and endothelial cells, thereby initiating coagulation cascades, and leading to fibrin formation [40]. As part of the extrinsic pathway, thrombin, plasmin and other enzymes can directly cleave C5 to C5a, leading to microangiopathy with thrombosis [14,35]. Furthermore, C5a promotes the formation of neutrophil extracellular traps (NETs), bearing functional TF, further potentiating local coagulation processes [35]. NETs are produced by activated neutrophils and consist of DNA, histones, granular proteins such as neutrophil elastase, cathepsin G, and myeloperoxidase [42]. In ARDS, the aggregation and infiltration of neutrophils in the lungs have a significant influence on the development of the disease [43]. Neutrophils regulate inflammatory responses through various pathways, and the release of NETs from neutrophils is considered to be one of the most important mechanisms [44].

## 4. C5a and C5aR1 in Sepsis and ARDS

Dysregulation of the C5a/C5aR1 signaling axis plays a central pathophysiological role in the development and progression of sepsis and ARDS by impairing host defense mechanisms, promoting tissue damage, and driving coagulopathy [22,45]. There is a wealth of pre-clinical and clinical evidence that demonstrates the important role of C5a–C5aR signaling in sepsis and ARDS.

### 4.1. Pre-Clinical Evidence

The critical role of C5a–C5aR1 signaling in sepsis has been demonstrated across multiple pre-clinical sepsis models. There is strong evidence for complement activation in animal models of sepsis, as reflected by elevated plasma levels of C3a, C4a, and C5a [46,47], and complement has been shown to be activated across multiple pathways [22,48,49], including the extrinsic pathway [50].

Studies conducted almost 40 years ago in a primate model of sepsis, for example, demonstrated that blockade of C5a by antibodies could improve outcomes following infusion of *Escherichia coli*, which induced septic shock and ARDS in monkeys [51,52]. In more recent studies in baboons with *E. coli* sepsis, pre-treatment with the C5 cleavage inhibitor RA101295 significantly reduced organ damage and mortality [53]. These findings in primates are supported by experiments in a rodent model of lipopolysaccharide (LPS)-mediated sepsis. LPS-induced septic shock could be mimicked by administration of C5a, and inhibition of C5a with neutralizing antibodies reduced the hypotensive response to LPS and the development of septic shock in this model [47]. Additionally, a number of studies have used the rodent model of sepsis following cecal ligation and puncture (CLP) to show that blockade of either C5a or C5aR1 (using IgG antibodies or a C5aR1 antagonist) leads to significant improvements in survival rates. This model of sepsis closely mimics the pathophysiology of sepsis in humans and remains the gold-standard rodent model for experimental polymicrobial sepsis [54,55]. In the CLP model, excessive activation of complement and generation of C5a leads to loss of neutrophil chemotaxis, phagocytic ability, and oxidative burst function, accompanied by diminished C5aR1 expression on neutrophils, systemic immune dysregulation, and decreased bacterial killing capacity. While C5a initially drives strong neutrophil activation, persistent overactivation caused by excessive C5a leads to neutrophil exhaustion, ultimately impairing their antimicrobial and effector functions. This functional paralysis contributes to immune dysregulation and worsens outcomes in sepsis and related conditions. Experiments in which these animals have been treated with anti-C5a or anti-C5aR1 antibodies have shown significantly improved neutrophil function and survival [56,57]. Further studies have demonstrated that the use of anti-C5a antibodies in the CLP group can preserve neutrophil function, reduce bacterial burden in blood and organs, and improve survival (compared with the reference group) [58]. Anti-C5a treatment can also prevent the development of multiorgan failure and the accompanying onset of blood neutrophil dysfunction [59]. Use of the CLP model also demonstrated that C5aR1 is upregulated in lung, liver, kidney, and heart during the early phases of sepsis, and that anti-C5aR1 treatment significantly improved survival [60]. In addition to decreasing mortality in bacterial sepsis induced by CLP in rodents, anti-C5aR1 treatment can reduce mortality in both the H5N1-induced viral sepsis model and malaria-induced cerebral sepsis model in mice [61]. Furthermore, in a mouse model of MERS-CoV-induced viral pneumonia, treatment with an anti-C5aR1 monoclonal antibody improved lung pathology and reduced viral titer [62]. Taken together, preclinical evidence across multiple models underscores that C5a is a pivotal contributor to sepsis pathophysiology. Inhibiting C5a/C5aR1 interactions consistently confers protective effects.

### 4.2. Clinical Evidence

Human studies have shown that complement activation and elevated levels of C5a are associated with sepsis and/or ARDS. Increased complement activation and elevated C5a levels have been reported in patients at risk of developing ARDS due to bacteremia or trauma [63]. A study of patients admitted to the intensive care unit (ICU) also demonstrated that complement activation and elevated C5a-like activity was associated with ARDS [64]. A further study that assessed complement activation in both plasma and bronchoalveolar lavage fluid obtained from patients with ARDS showed activation of C3 and C5a in their epithelial lining fluid and suggested that C5a may account for the large influx of neutrophils into the lung seen in ARDS [45]. More recently, a longitudinal analysis of immune responses in patients with various stages of COVID-19 found an increase in soluble C5a levels associated with COVID-19 severity, and high levels of C5aR1 expression in blood and pulmonary myeloid cells, supporting a role for the C5a-C5aR1 axis in the pathophysiology of ARDS [65].

In a study of 27 patients with sepsis in whom C3a and C5a were found to be elevated on hospital admission, successful treatment of sepsis returned C3a and C5a levels to normal within one week, while persistently elevated levels were observed in patients who developed multisystem organ failure, suggesting that sustained high levels of C3a and C5a are associated with worse clinical outcomes [66]. C5a serum levels have been found to be related to sepsis severity, with C3a, C4a and C5a significantly higher in patients who died of sepsis compared with survivors [67]. Complement activation in patients with sepsis has also been documented with plasma concentrations of the terminal complement complex (MAC; C5b-9) found to increase by an average of 110% two days prior to the onset of ARDS in septic patients [68]. Studies using C5a-neutralizing L-RNA-aptamers have provided further insight into the role of C5a in inflammatory lung conditions. Neutralization of C5a using an L-RNA aptamer in a human whole blood model of meningococcal sepsis significantly inhibited CD11b/CD18 (CR3) upregulation, phagocytosis, and oxidative burst, while preserving MAC formation and complement-mediated bacterial killing [69]. This indicates that anti-C5a therapy can attenuate harmful inflammatory responses without impairing host defense mechanisms that depend on the MAC.

The observation of complement-mediated neutrophil dysfunction is another important clinical finding. Barrett et al. found that hemodynamic shock primes neutrophils for extracellular ROS production in a C5a-dependent manner, contributing to endothelial barrier loss and organ injury [70]. Complement activation has also been reported to be associated with enhanced thrombotic activity, and blockade of C5aR1 can alleviate platelet-mediated thrombogenicity in a NETs-dependent manner in an ex vivo experiment using plasma from COVID-19 patients [35]. Silva et al. also found that C5aR1 signaling drives NETs-dependent immunopathology in COVID-19 patients [71]. Increased NETs formation is associated with microthrombus and platelet accumulation in the pulmonary circulation, indicating that NETs promote thrombosis in ARDS [72]. High expression levels of C3a, C5a, IL-8 and RANTES have been found in the lungs of MERS-CoV-infected patients. Finally, the upregulation of lung C5a and C3a was positively correlated with IL-8, RANTES, and fatality rate [73].

Clinical studies across diverse infectious and inflammatory conditions consistently demonstrate that C5a generation and C5aR1 signaling play a central role in driving systemic inflammation, thrombosis, neutrophil dysfunction, and multiorgan failure in sepsis and ARDS.

## 5. Upstream Complement Inhibitors That Do Not Fully Block C5a

Based on the wealth of preclinical and clinical evidence on the role of complement and activation of the C5a/C5aR1 axis in sepsis and ARDS, pharmacological targeting of the complement system with inhibitors of complement cascade proteins has considerable promise in the treatment of these, and potentially other inflammatory diseases.

Eculizumab is a C5 inhibitor which prevents the generation of C5a from conventional complement pathways and prevents the formation of the MAC [74]. While eculizumab has proven effective in treating rare complement-mediated diseases like paroxysmal nocturnal hemoglobinuria (PNH) and atypical hemolytic uremic syndrome (aHUS), there have been mixed findings in COVID-19. For example, in a pediatric case of severe COVID-19-associated ARDS, eculizumab treatment was associated with suppression of MAC formation, but did not lead to a corresponding decrease in C5a levels [75]. Despite this, some small-scale studies reported favorable outcomes with eculizumab. A case series involving four adult patients with severe COVID-19 treated with eculizumab documented clinical recovery in all cases [76]. Similarly, a proof-of-concept study showed a higher 15-day survival rate among ICU patients treated with eculizumab compared with standard of care (SOC) (82.9% vs. 62.2%) [74]. However, the same study reported a significantly higher incidence of ventilator-associated pneumonia and a numerically increased rate of bacteremia in the eculizumab arm [74]. Similar safety concerns were found with another C5 inhibitor, ravulizumab. In the Phase 3 trial of ravulizumab in non-invasive ventilated or IMV patients (n = 202), no survival benefit was observed compared with best supportive care [77]. Moreover, patients receiving ravulizumab had nearly twice the incidence of infections and vascular complications [77]. Similarly, the Phase 4 TACTIC-R trial found no differences in mortality or time to ventilation between ravulizumab plus SOC, baricitinib plus SOC and SOC alone, leading to early termination due to futility [78]. Progression of COVID-19 pneumonia was twice as high in the ravulizumab and baricitinib groups compared with SOC alone. The authors noted that the therapeutic potential of targeting C5a, rather than only the inhibition of C5, warranted further evaluation. Interestingly, despite the fact that complement pathways converge at C3, the C3 inhibitor APL-9 (NCT04402060) also failed to significantly reduce mortality in COVID-19–associated ARDS [79].

These clinical findings support earlier preclinical studies suggesting that targeting C5a specifically, rather than other upstream components of the complement system, may be preferable in the treatment of inflammatory diseases such as severe COVID-19 leading to ARDS.

## 6. Vilobelimab

### 6.1. Mechanism of Action

Vilobelimab is a first-in-class, targeted C5a antibody which blocks the biological activity of C5a in human blood through strong and highly selective binding to C5a (Figure 1). By inhibiting C5a, vilobelimab prevents its interaction with C5aR1, thus potentially blocking downstream effects including neutrophil activation, cytokine storm, endothelial dysfunction and immunothrombotic responses [23,80]. Unlike upstream C5 inhibitors, vilobelimab does not interfere with the formation of C5b or the MAC, thus preserving the complement system’s essential bactericidal function [18,80,81]. In addition to preserving the MAC, the rationale for specifically targeting C5a with vilobelimab is supported by evidence that C5a can be generated independently of conventional complement convertases via cleavage by extrinsic proteases such as thrombin and trypsin [18]. This means that C5 inhibitors (e.g., eculizumab), while effectively blocking C5 cleavage by convertases, fail to inhibit C5a generated from direct enzymatic cleavage, leaving a significant amount of biologically active C5a that continues to drive inflammation [18]. In contrast, vilobelimab specifically binds and inhibits free C5a, including C5a generated via the extrinsic pathway.

### 6.2. Preclinical Evidence

C5a inhibition with an anti-C5a blocker such as vilobelimab has been shown to improve outcomes in both infective- and chemical-induced pre-clinical ARDS models. Direct inhibition of C5a with a different anti-C5a antibody in mice infected with H5N1 significantly reduced lung tissue damage (*p* < 0.001) and improved survival (*p* < 0.01) compared with controls [82]. In the African green monkey model of H7N9 avian viral pneumonia, vilobelimab (also known as IFX-1) significantly reduced neutrophilic inflammation, lung injury, systemic cytokine release, and viral load [83]. In a non-human primate model of paraquat induced lung injury, vilobelimab improved lung injury marker scores while also preventing lung tissue damage with significant reductions in neutrophils, macrophages, and systemic inflammatory responses [84].

### 6.3. Clinical Evidence in Sepsis

The Phase IIa randomized SCIENS trial evaluated vilobelimab in patients with early severe sepsis or septic shock [80]. In this study conducted across 11 German ICUs, patients were randomly assigned in a ratio of 2:1 to three subsequent dosing cohorts for IV vilobelimab or placebo receiving either 2 × 2 mg/kg (0 and 12 h), 2 × 4 mg/kg (0 and 24 h) or 3 × 4 mg/kg (0, 24, and 72 h). Vilobelimab led to a significant, dose-dependent reduction in plasma C5a levels, achieving up to a 90% reduction, sustained for over five days in the highest dosing group. Importantly, the treatment did not impair MAC formation (CH50 remained stable), confirming its selectivity for C5a. Vilobelimab was well-tolerated across all dose cohorts. No treatment-related safety signals or immunogenicity concerns were observed. In the overall study, patients receiving the two higher doses of vilobelimab experienced more ICU-free [19.5 (6–24) and 12.5 (0–23)] and ventilator-free days [26.5 (13–27) and 24.5 (3–28)] until day 28, respectively, compared with the lower dose [5.0 (0–18) ICU-free days, 10 (0–27) ventilator-free days] as well as fewer ICU-free days compared with placebo [2.5 (0–20)]. Cytokine analyses indicated faster reductions in IL-8 and IL-10 levels among vilobelimab-treated patients compared with placebo, suggesting a reduction in inflammatory burden and the ability of vilobelimab to attenuate the hyperactive immune response and restore immune balance. IL-10 is generally considered an anti-inflammatory cytokine and its increase along with other cytokines in sepsis and ARDS serves as a warning signal of increased inflammation [85]. In the context of treatment of early severe sepsis or septic shock with vilobelimab, decreasing IL-10 levels suggests that inflammation is being reduced by the anti-C5a monoclonal antibody in these patients. Although not powered for mortality endpoints, the trial provided strong support for further clinical investigation.

### 6.4. Clinical Efficacy and Safety in COVID-19 Induced ARDS

The Phase II PANAMO study comparing vilobelimab plus best SOC with best SOC alone was performed at three sites in the Netherlands in critically ill patients with COVID-19–induced ARDS (n = 30) [81]. Vilobelimab significantly reduced serum C5a levels from a median of 189.98 ng/mL (22.9 nM) to 39.70 ng/mL (4.8 nM) after a single dose. This reduction was sustained through day 8, while C5a levels in the placebo group remained elevated (158.53 ng/mL; 19.1 nM; *p* = 0.0006) [24]. Clinical outcomes favored vilobelimab, with 28-day mortality at 13% versus 27% in the best SOC alone group. Fewer pulmonary embolisms and infections were reported in the vilobelimab arm.

Building on the Phase II proof-of-concept study, the international, multicenter, Phase 3, double-blind, placebo-controlled PANAMO study randomized critically ill, moderate-to-severe patients with COVID-19–induced ARDS to vilobelimab plus SOC (n = 177) or placebo plus SOC (n = 191) [25]. SOC consisted of corticosteroids (97%), anti-coagulant prophylaxis (98%) and other immunomodulators (~20% tocilizumab > baricitinib). All-cause mortality in the vilobelimab plus SOC group was ~32% vs. ~42% in the placebo + SOC group, and the intent-to-treat analysis (non-site stratified) showed a significant reduction in mortality compared with placebo [Hazard Ratio (HR) 0.67, 95% CI 0.48–0.96; *p* = 0.027). The site-stratified analysis, recommended by the FDA for the Phase 3 study, showed the same reduction in absolute mortality but did not reach statistical significance (HR 0.73, 95% CI 0.50–1.06; *p* = 0.094). This was attributed to the exclusion of 61 patients from the Cox regression analysis (the inherent algorithm ‘ignores’ data from 55 patients from sites with no events or deaths, and 6 patients who died from single patient enrollment sites, all of whom were in the placebo group) [86]. However, during the approval process, the FDA recognized the issue with the site stratified Cox regression analysis and agreed that a reversion to the originally proposed non-site stratification can be used as it stays true to the intent-to-treat principle. This led to the issuance of the EUA in the United States [87]. In addition, vilobelimab was approved by the EMA for the treatment of COVID-19 induced ARDS [88]. Adverse events were generally similar between groups with more pneumonia and septic shock in the vilobelimab plus SOC group, essentially due to these patients surviving longer than those in the placebo plus SOC group [25]. Infections were similar between arms, with numerically more fungal (n = 8) and viral (n = 6) infections in the vilobelimab plus SOC than placebo plus SOC groups, which investigators ascribed to nosocomial infections, also likely related to surviving longer in ICU on IMV. A series of post hoc analyses of this study explored the potential benefits of combination therapy with vilobelimab and IL-6 inhibition [89,90,91]. These analyses demonstrated that dual immunomodulation in the subgroup where tocilizumab or baricitinib was also administered, targeting both the C5a/C5aR1 axis and IL-6 signaling, was associated with improved mortality in critically ill patients with COVID-19, with no significant safety signals. One sub-analysis (n = 61) showed that among patients receiving both vilobelimab and tocilizumab, 28-day all-cause mortality was reduced to 7.1% compared with 42.3% in those receiving placebo and tocilizumab (HR 0.14, 95% CI 0.03–0.62; *p* = 0.010) [89]. At day 60, mortality was 18.9% versus 49.1%, respectively (HR 0.30, 95% CI 0.11–0.82; *p* = 0.019). This presumed synergy could be explained by a previously described cross-talk between IL-6 and the C5a/C5aR1 axis in pre-clinical sepsis studies, which found that IL-6 induces C5aR1 expression during sepsis in various tissues and C5a in turn induces IL-6 upregulation in certain immune cells [89,92,93]. Similarly, it is known that C5a binding to C5aR1 also induces STAT3 expression suggesting an interplay between C5a and inflammatory stimulation of cytokine generation via the JAK/STAT pathway [94,95]. Importantly, the combination of vilobelimab and tocilizumab was well-tolerated [89,90,91].

## 7. Conclusions and Future Perspectives

A wealth of preclinical and clinical studies has shown that C5a is generated at a high level in sepsis and ARDS of differing etiologies and is a key driver of pathology. Building on this evidence, vilobelimab was designed to selectively neutralize C5a, and prevent subsequent C5a-induced inflammation. Unlike upstream complement inhibitors, vilobelimab blocks C5a generated through both the extrinsic pathway as well as conventional complement pathways, while also allowing C5b to be generated and the associated MAC formation to remain intact. This desirable mechanism of action has been shown to translate into meaningful clinical outcomes, with vilobelimab being the only drug to demonstrate a significant reduction in mortality in patients with COVID-19–induced ARDS requiring IMV and ECMO in a properly powered and placebo-controlled study, with safety comparable to placebo. Given the demonstrated efficacy and safety of vilobelimab in the management of COVID-19–induced ARDS in the PANAMO study, results of the ongoing BARDA study (NCT06703073) in a broader patient population with ARDS are awaited.
